# Surgical Management of Congenital Lung Malformations in Children—A Single‐Center Analysis of 25 Years of Experience

**DOI:** 10.1111/crj.70178

**Published:** 2026-02-23

**Authors:** Patrycja Sosnowska‐Sienkiewicz, Alicja Kamińska, Przemysław Mańkowski, Irena Wojsyk‐Banaszak, Danuta Januszkiewicz‐Lewandowska

**Affiliations:** ^1^ Department of Pediatric Surgery Medical University of Warsaw Warsaw Poland; ^2^ Department of Pediatric Oncology, Hematology and Transplantology Poznan University of Medical Sciences Poznan Poland; ^3^ Department of Pediatric Surgery, Traumatology and Urology Poznan University of Medical Sciences Poznan Poland; ^4^ Department of Pulmonology, Pediatric Allergy and Clinical Immunology Poznan University of Medical Sciences Poznan Poland

**Keywords:** bronchogenic cyst, bronchopulmonary sequestration, congenital cystic adenomatoid malformation, congenital lobar emphysema, congenital lung malformations, congenital pulmonary airway malformation, pulmonary agenesis, pulmonary sequestration, surgical treatment

## Abstract

**Introduction:**

Congenital lung malformations (CLMs) in pediatric patients encompass various structural abnormalities arising during fetal development, which can range from benign to life‐threatening. The most common types include congenital pulmonary airway malformation (CPAM) and bronchopulmonary sequestration (BPS).

**Aim of the Study:**

This study aimed to retrospectively analyze patients treated surgically for CLMs, focusing on indications for surgery, surgical techniques, and outcomes.

**Materials and Methods:**

Data were collected from the medical records of patients who underwent thoracoscopy (*n* = 140) or thoracotomy (*n* = 52) between 2000 and 2024. Among these, 50 patients were diagnosed with CLMs, who were taken for further analysis. Study group inclusion criteria were performing a CT/X‐ray imaging examination indicating the presence of a defect, surgery, and available pathology results. Exclusion criteria were incomplete data or lack of surgical procedure. Final study group included 37 patients who met inclusion criteria for further analysis. Detailed analysis encompassed demographics, clinical presentation, diagnostic methods, treatment, and follow‐up.

**Results:**

The cohort included patients diagnosed with CPAM type I (*n* = 12), CPAM type II (*n* = 7), pulmonary sequestration (*n* = 10), and other congenital malformations such as bronchogenic cyst (*n* = 2), congenital cystic pulmonary disease (*n* = 2), CPAM type IV—pleuropulmonary blastoma type I (PPB) (*n* = 1), juvenile emphysema (*n* = 2), and mediastinal cyst (*n* = 1). The average age at diagnosis was 37.61 months. The cohort consisted of 17 females and 20 males. The right lung was involved in 41.18% of cases, and the left lung in 58.82%. Symptoms at presentation included pneumonia (*n* = 9), respiratory failure (*n* = 8), emphysema (*n* = 3), and pneumothorax (*n* = 2). Fifteen patients were asymptomatic, and the diagnosis was incidental. Seven patients had other congenital diseases, such as heart defects. None of the patients other than the child with PPB were offered genetic diagnostics, albeit for DICER1 or KRAS mutations.

**Conclusion:**

The study underscores the heterogeneity in age and clinical presentation at the time of CLM diagnosis, highlighting the importance of an individualized and tailored approach to management.

## Graphical abstract

Early diagnosis and intervention are critical for improving prognosis in children with congenital lung malformations (CLM). Fetal diagnosis of CLM generally leads to favorable outcomes; however, there is limited evidence on the effectiveness of fetal therapy in improving outcomes for these anomalies. Collaborative management involving multiple specialties is essential for optimal outcomes.

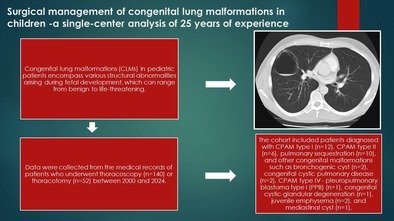

## 1 Introduction

Congenital lung malformations (CLMs) in pediatric patients include a variety of structural abnormalities that occur during fetal development. These malformations can range from relatively benign to life‐threatening conditions [1].

Congenital pulmonary airway malformation (CPAM), also known as congenital cystic adenomatoid malformation (CCAM), involves abnormal lung tissue development, resulting in cysts or solid masses in the lung. Historically, CPAMs were classified by the Stocker classification, defined by Roman numerals—type I were macrocystic, type II were mixed, and type III were solid/microcystic [2]. Pathologists sought more precise terminology for these lesions, many of which were neither cystic nor adenomatous. Based on the association with abnormal histology in relation to the major airways, there are five different types of CPAM, defined by Arabic numerals (0–4) [3]. The underlying cause for CPAM is not known. The association between CPAM and malignancy was observed. There is a risk of about 0.7% of malignant transformation within the cyst [4]. *KRAS* has already been confirmed to be somatically mutated in CPAM, and other genetic susceptibilities linked to tumor development have been explored. There are a variety of genes implicated in CPAM, including DICER1, thyroid transcription factor gene (Nkx2), platelet‐derived growth factor B gene (PDGF‐B), transforming growth factor B (TGFB), and fibroblast growth factors 10, 9, and 7 (FGF10, 9, 7) [3].

Bronchopulmonary sequestration (BPS) involves nonfunctioning lung tissue that does not communicate with the normal airway. It can either be intralobar (within the lung, 75%) or extralobar (outside the lung, 25%). CPAM and BPS are often confused with each other because they both produce a mass effect and tend to affect the lower lobes [5]. One study suggests that one‐quarter of lung samples have histology consistent with CPAM but are also supplied by a systemic arterial blood supply (i.e., hybrid/mixed lesions) [6].

Bronchogenic cysts (BC) are fluid‐filled sacs arising from abnormal budding of the primitive tracheobronchial tree. Based on imaging studies, bronchial cysts are usually located more centrally in the chest. Large, enlarging BC may cause central airway obstruction, also with distal pulmonary hyperinflation [7, 8].

Pulmonary agenesis (PA) involves the complete absence of lung tissue in one or both lungs. It can relate to oligohydramnios and/or fetal anomalies in prenatal ultrasound examination. PA may be associated with Potter's syndrome, fetal renal anomalies, and preterm premature rupture of membranes [6].

Congenital lobar emphysema (CLE) is a rare disorder associated with acute respiratory failure in newborns. Antenatal diagnosis is rare due to its echogenic appearance and lack of mediastinal shift in utero [9]. CLE is prone to overdistension of the affected parenchyma for a variety of reasons, which may include internal bronchoconstriction, bronchomalacia, external bronchoconstriction, and multilobular lobe [10]. Bronchial atresia (BA) is an absence or narrowing of a segment of a mainstem or lobar bronchus [6].

The incidence of CLMs varies depending on the type and population studied. Their incidence has increased in recent years based on several population‐based studies, which suggest a frequency of 1 in 2500 live births [1, 6]. CPAM is the most common, with an estimated incidence of 1 in 11 000 to 1 in 35 000 live births. Large‐cyst subtypes account for approximately 70% of CPAMs, or 2 to 8 per 100 000 live births [11, 12].

Symptoms may vary widely depending on the size, location, and type of malformation, but they can include respiratory distress, persistent cough, recurrent respiratory infections, chest pain, wheezing, failure to thrive, cyanosis (bluish discoloration of the skin), or abdominal mass (in cases of extralobar sequestration) [6].

CLMs can be diagnosed in different ways. During routine prenatal ultrasound examinations, some lesions may be detected. Approximately 70% of all CLMs are currently detected during fetal anatomical examination [13]. An abnormal chest X‐ray may prompt further investigation, the same ultrasound examination of the chest or abdomen. Computed tomography and magnetic resonance provide detailed information about the size, location, and characteristics of the malformation. Bronchoscopy is also the way for direct visualization of the airways [1, 6]. Pulmonary function tests may be performed to assess lung function and capacity [5, 6].

The treatment approach depends on various factors, including the type and severity of the malformation, as well as the patient's clinical condition. Treatment options may include observation in the case of small, asymptomatic CLMs. In these cases, they can be monitored without intervention. The role and timing of surgery in asymptomatic lung lesions remain controversial [14]. Surgical resection may be necessary for symptomatic or composed malformations. Minimally invasive procedures such as thoracoscopic or laparoscopic resection may be used. In extensive cases, thoracotomy or laparotomy, depending on lesion localization, is performed. Patients may need respiratory support, antibiotics, and nutritional additional therapy [6]. Genetic counseling is currently required.

The prognosis for CLMs is generally good with appropriate management. Many children have complete resolution of symptoms after surgical resection, especially if the malformation is identified and treated early. However, the prognosis can vary depending on the size, type, and associated complications of the malformation. Close monitoring and follow‐up are essential to ensure optimal outcomes [5, 6].

## 2 Aim of the Study

The purpose of this study was to retrospectively analyze patients treated surgically for CLMs, including indications for surgery, surgical techniques used, and results obtained.

## 3 Materials and Methods

**FIGURE 1:**
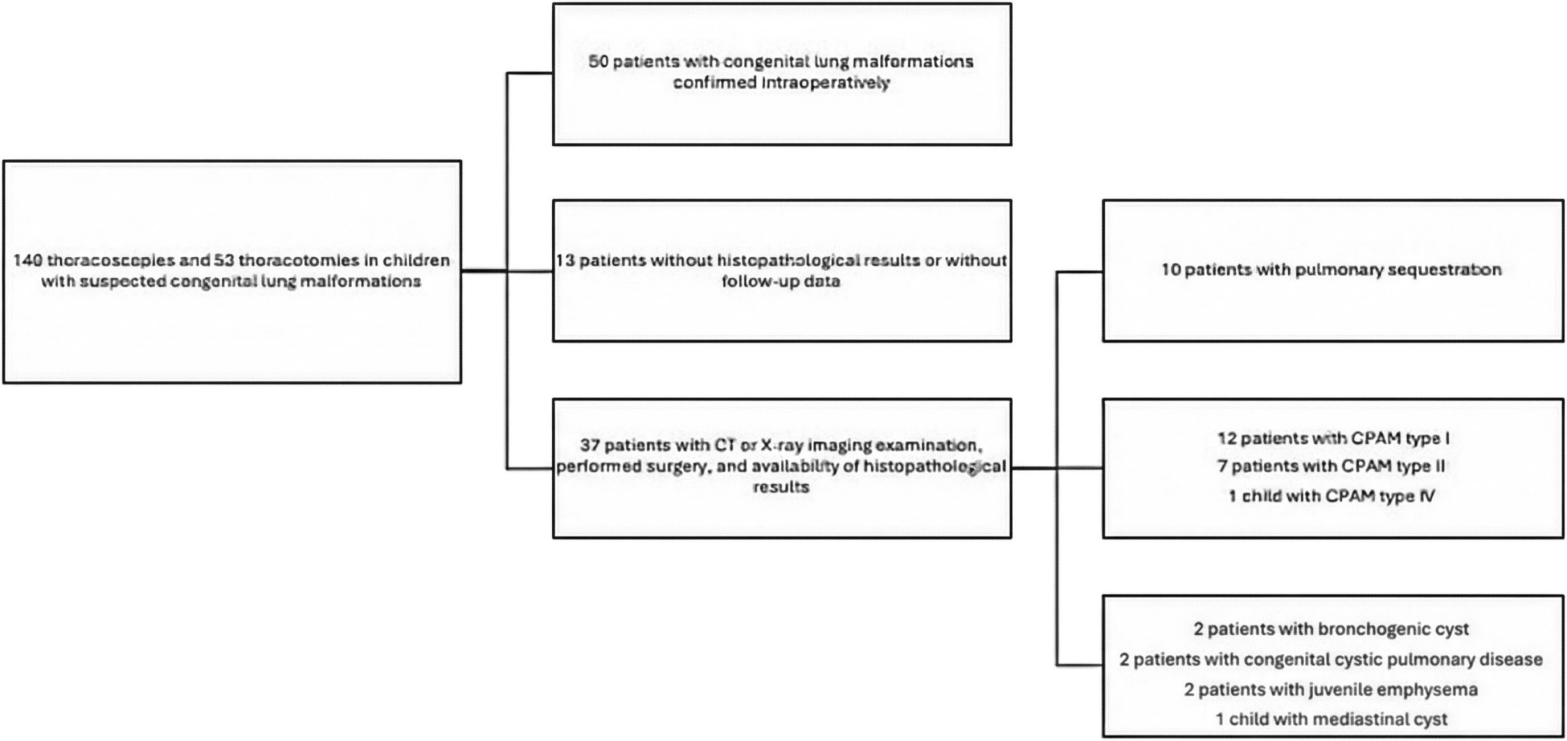
A diagram illustrating the inclusion and exclusion of patients in the study.

Data were collected from the medical records of patients treated in the Department of Pediatric Surgery, Traumatology, and Urology or in the Department of Pediatric Pneumonology, Allergology, and Clinical Immunology at the Poznan University of Medical Sciences from 2000 to 2024. Over the 25‐year period, 140 thoracoscopies and 52 thoracotomies were performed in children with suspected CLMs. Among them, CLMs were confirmed intraoperatively in only 50 patients, who were subsequently included for further analysis. The inclusion criteria for that group were a CT or X‐ray imaging examination indicating the presence of a lesion, a performed surgical procedure, and the availability of histopathological results. The exclusion criteria included incomplete data or lack of histopathological examination. The final study group consisted of 37 patients who met the inclusion criteria for further analysis. A diagram illustrating the inclusion and exclusion of patients in the study is presented in Figure 1.

Statistical analysis was performed using Microsoft Excel. Continuous variables are presented as means with standard deviation (SD) and range (min–max). Categorical variables are presented as counts and percentages. Comparisons between groups were performed using the Mann–Whitney *U* test for non‐normally distributed continuous data (age) and Fisher's exact test for categorical data. A *p*‐value of < 0.05 was considered statistically significant.

Among the study group, detailed analysis included age, gender, comorbidities, preliminary diagnosis, family history, including DICER 1 mutation, course of pregnancy (complicated, prenatal diagnosis, and type of delivery), symptoms before diagnosis (cough, pneumonia/bronchitis, and others), the way of lesion detection (accidentally, in the course of a lower respiratory tract infection, and other), final histopathological diagnosis, treatment: conservative/surgery, age of diagnosis and surgery, diagnostics (ultrasound, KT, and MR), baseline lesion size, location, age of last follow‐up/observation, size of lesion during follow‐up (if observation), problems in the child follow‐up (lower respiratory tract infections and other), procedure (thoracoscopy/thoracotomy, time of surgery, and complications), postoperative period (complications and need for additional therapy). Each patient underwent surgical evaluation 7–10 days and 3 months if operative treatment was performed. All patients remained under constant follow‐up by a pulmonologist and/or oncologist.

Microsoft Excel was used for data collection. The study was approved by the Bioethics Committee of the Poznan University of Medical Sciences (Resolution no. KB‐280/24, April 5, 2024). All parents/legal guardians of patients involved had provided their informed consent prior to inclusion in the study.

## 4 Results

The cohort included patients diagnosed with pulmonary sequestration (*n* = 10; 27.0%), CPAM type I (*n* = 12; 32.4%), CPAM type II (*n* = 7; 18.9%), and other congenital malformations, including BC (*n* = 2; 5.4%), congenital cystic pulmonary disease (*n* = 2; 5.4%), juvenile emphysema (*n* = 2; 5.4%), CPAM type IV (pleuropulmonary blastoma [PPB] type I) (*n* = 1; 2.7%), and mediastinal cyst (*n* = 1; 2.7%). Among the 10 patients diagnosed with pulmonary sequestration, seven were identified with extrapulmonary sequestration and two with intrapulmonary sequestration. One case was indeterminate due to insufficient diagnostic clarity. CPAM type I was the most prevalent (32.4%), followed by type II (18.9%).

The average age at diagnosis was 37.61 (SD ± 56.27; range: 0–198 months) months. The cohort consisted of 17 females and 20 males. The cohort consisted of 17 females (45.9%) and 20 males (54.1%). The average age at diagnosis for females was 27.82 months (SD ± 48.05), while for males, it was 45.90 months (SD ± 62.43). Statistical analysis showed no significant difference in age distribution between genders (*p* > 0.05). The average age at diagnosis for CPAM (I + II) was 25.8 months. For pulmonary sequestration, the average age at diagnosis was 67.09 months. The right lung was involved in 41.18% of cases, and the left lung in 58.82% of cases. Among the lobes, the superior lobe was affected in 46.67% of cases, the inferior lobe in 50.00% of cases, and the middle lobe in 3.33% of cases. Only four patients were diagnosed prenatally. The presenting symptoms included pneumonia (*n* = 9), respiratory failure (*n* = 8), emphysema (*n* = 3), and pneumothorax (*n* = 2). Fifteen patients were asymptomatic (Table 1).

**TABLE 1:** Detailed symptom analysis in CPAM group.

Symptom/type of CLMs	CPAM I	CPAM II	CPAM IV	BPS	Others
	*N* = 12	*N* = 6	*N* = 1	*N* = 10	*N* = 8
Pneumonia	3	1	1	0	4
Respiratory failure	2	2	0	1	3
Emphysema	2	0	0	0	1
Pneumothorax	1	0	0	0	1
Asymptomatic	4	3	0	9	0

*Note:* Others including bronchogenic cyst (*n* = 2), congenital cystic pulmonary disease (*n* = 2), congenital cystic‐glandular degeneration (*n* = 1), juvenile emphysema (*n* = 2), and mediastinal cyst (*n* = 1).

Abbreviations: BPS, bronchopulmonary sequestration; CPAM, congenital pulmonary airway malformation.

Seven children had other congenital diseases. These included atrial septal defect (ASD) II, ventricular septal defect (VSD), prematurity, twin pregnancy, atopic nodal tachycardia, hypothyroidism, aorta with a left‐sided arch, foramen ovale, pericardial fluid, galactosemia, and multilocular cystic nephroma. In our subgroup, we have different types of congenital lung diseases with no predilection to one of them (Table 2).

**TABLE 2:** Characteristics of the coexistence of congenital lung malformations with other congenital diseases in seven patients.

Sex	Type of congenital lung malformation	Symptoms at birth	Age at the time of diagnosis [months]	Lung affected	Lobe affected	Size of the malformation	Surgical resection	Other congenital diseases
F	Sequestration	Asymptomatic	1	Left	Inferior	7 × 5 × 3 cm	Thoracotomy	ASD II, VSD
M	CPAM I	Respiratory failure	2	Right	Superior	4.5 × 3 × 2 cm	Thoracotomy	ASD II, atypical nodal tachycardia, hypothyroidism
M	CPAM II	Respiratory failure	2	Right	Superior	9 × 7 × 3 cm	Lobectomy	Left‐sided aortic arch
M	Emphysema	Emphysema	2	Right	Superior	8.5 × 8.2 × 2.4 cm	Thoracotomy	Foramen ovale, galactosemia
F	CPAM IV (pleuropulmonary blastoma type I)	Pneumonia	16	Right	Superior	6 × 4 × 3 cm	Lobectomy	Multilocular cystic nephroma
M	CPAM II	Respiratory failure	0	Left	Superior	7 × 5 × 3 cm	Thoracotomy	ASD II
F	CPAM I	Pneumothorax	11	Left	Inferior	8.5 × 7 × 4 cm	Thoracotomy	PDA

The course of the surgical procedure was favorable for all patients. Periodic pulmonological check‐ups were conducted over a period of 1 year. None of the patients experienced long‐term complications after surgery, and those included chronic cough, recurrent lower‐airway infections, wheezing, poor tolerance to exercise, or orthopedic complications. No recommendations for strict oncological follow‐up were given to any of the patients. None of the patients had genetic testing for the DICER1 mutation recommended. All patients are alive. In one patient, the change turned out to be cancerous, and the child needed chemotherapy.

Surgical indications included respiratory distress (*n* = 8; 21.6%), recurrent pneumonia or infections (*n* = 12; 32.4%), progressive lesion growth on imaging (*n* = 7; 18.9%), pneumothorax (*n* = 2; 5.4%), suspicion of malignancy (*n* = 1; 2.7%), and prenatal diagnosis with expected postnatal complications (*n* = 7; 18.9%). Thoracoscopy was the primary approach, with thoracotomy used for large lesions, complex anatomy, or conversion. Lobectomy was the standard procedure for CPAM and congenital emphysema, while sequestration and cystic lesions were excised completely when anatomically feasible. The choice of procedure (thoracoscopy vs. thoracotomy and lobectomy vs. lesion excision) was based on lesion type, anatomical complexity, and surgeon's discretion, consistent with current clinical standards (Table 3).

**TABLE 3:** Surgical indications and procedures by CLM type.

CLM type	*n*	Main indications for surgery	Surgical procedure	Surgical approach
**CPAM type I**	12	Symptomatic respiratory distress; recurrent pneumonia; progressive enlargement on imaging; pneumothorax (selected cases); prenatal diagnosis with postnatal risk	**Lobectomy (majority), segmentectomy in peripheral lesions**	Thoracoscopy preferred; thoracotomy for large/complex lesions or conversion
**CPAM type II**	7	Recurrent infections; respiratory distress; progressive lesion	**Lobectomy; segmentectomy (selected small lesions)**	Thoracoscopy preferred
**CPAM type IV/PPB type I**	1	Suspicion of malignancy; pneumothorax	**Lobectomy + oncologic follow‐up**	Thoracotomy
**Pulmonary sequestration (total)**	10	Symptomatic cases: respiratory infections, respiratory failure; asymptomatic extrapulmonary lesions elective resection; diagnostic uncertainty	**Extrapulmonary sequestration: complete lesion excision; Intralobar: lobectomy or segmentectomy**	Thoracoscopy preferred; thoracotomy in large lesions or difficult vascular anatomy
‐Extralobar sequestration	7	As above, plus mass effect risk	Excision of lesion with feeding vessel control	Thoracoscopy
‐Intralobar sequestration	2	Recurrent infections	Lobectomy (standard)	Thoracoscopy/thoracotomy
**Bronchogenic cyst**	2	Mass effect; risk of airway compression; infection; growth on imaging	Complete cyst excision	Thoracoscopy
**Congenital cystic pulmonary disease**	2	Recurrent infection or progressive lesion	Lobectomy/segmentectomy	Thoracoscopy
**Juvenile emphysema (congenital lobar emphysema)**	2	Neonatal/infant respiratory distress; hyperinflation; mediastinal shift	Lobectomy	Thoracotomy or thoracoscopy depending on respiratory stability
**Mediastinal cyst**	1	Compression risk; progressive growth	Cyst excision	Thoracoscopy

## 5 Discussion

CLMs represent a diverse spectrum of diseases, each with unique origins and a variety of symptoms, highlighting the importance of individualized management plans. Our cohort represents a diverse group of different CLMs, with CPAM being the prevailing one. In general, CPAM is divided into five subgroups based on cellular characteristics and cyst size. CPAM Type 0, the rarest (1%–3% of cases), originates from tracheal or bronchial tissue. Type 1 CPAM, the most prevalent form (60%–70% of cases), typically arises from the distal bronchi or proximal bronchioles and features thin‐walled cysts ranging from 2 to 10 cm in diameter. Type 2 CPAM constitutes 15%–20% of cases and is characterized by multiple small cysts (0.5 to 2 cm) that blend into the adjacent normal tissue, resembling dilated terminal bronchioles. Type 3 CPAM can involve an entire lung lobe or multiple lobes. Type 4 CPAM is strongly associated with malignancy, particularly PPB.

In our cohort, we did not observe CPAM Types 0 and 3 although one patient was diagnosed with PPB, proving the necessity for malignancy suspicion in infants presenting with pneumothorax and CPAM. Our data showed a predominance of CPAM Type 1 (12/19 patients) and CPAM Type 2 (7/19 patients), consistent with previous studies. All CPAM cases in our study had arterial and venous supply from pulmonary circulation, corroborating other studies that highlight the rarity of systemic circulation supply in CPAM. Notably, 40.5% of our patients were asymptomatic, while 22% presented with respiratory failure, aligning with literature reporting respiratory distress in 25%–30% of cases. The fact that the majority of our patients were asymptomatic is consistent with other studies showing that most of the patients did not develop any signs of these malformations [15].

Pulmonary sequestration was also observed in our cohort. PS is a rare congenital anomaly accounting for 1% to 6% of all pulmonary malformations at birth. It is characterized by a focal area of pulmonary tissue that lacks direct communication with the tracheobronchial tree and receives blood supply from systemic circulation rather than pulmonary circulation [16]. Our data showed that PS cases typically present at an older age compared to CPAM, likely due to less pronounced early‐life symptoms. Regarding gender distribution, our study included 17 females and 20 males. Although the average age at diagnosis appeared higher in males (45.90 months) compared to females (27.82 months), this difference was not statistically significant (*p* > 0.05), likely due to the wide age range in our cohort (0–198 months). Furthermore, statistical analysis confirmed no significant correlation between gender and specific malformation types. The variability in presenting symptoms in our cohort highlights the need for high clinical suspicion and comprehensive diagnostic workup in pediatric patients with respiratory issues, especially after birth. Computed tomography was the primary diagnostic tool used to distinguish CPAM from other pulmonary anomalies such as BPS, congenital diaphragmatic hernia, and CLE. CT or MRI remains critical for accurate diagnosis [17]. Approximately one‐third of CPAMs are diagnosed postneonatal period. If a prenatal CPAM diagnosis is made, these newborns should undergo chest radiographs, regardless of symptoms. Given the malignancy risk, all children should be evaluated with CT or MRI to classify the lesion and plan potential surgical removal. Management strategies should be tailored to the patient's symptoms. Symptomatic patients should undergo surgery, with the method chosen based on lesion localization and size. For asymptomatic patients, the decision between close observation and surgical resection remains debated [15]. The main argument for surgery is the elimination of future infection risks and higher radiation exposure due to more frequent CT scans as a part of patient's monitoring, potential malignant transformation and potential loss of follow‐up. Dossche et al. compared the incidence of lower respiratory tract infections among patients treated with conservative approach versus patients treated surgically. Among 217 patients, 81 (37%) undergoing surgery and 136 (63%) managed observationally. The incidence of lower respiratory tract infections (LRTI) did not significantly differ between the two groups at any follow‐up point. However, pre‐resection LRTI rates were significantly higher than post‐resection rates among children who underwent CLA‐related surgery [18]. For pulmonary sequestration, in symptomatic patients, surgical resection is recommended. However, in asymptomatic individuals with intralobar sequestration, surgical resection is not required but could be considered as prophylaxis to prevent recurrent infections. Asymptomatic individuals with extralobar sequestration, on the other hand, should undergo serial monitoring, as nonoperative management is appropriate [16]. As previous research shows, the optimal timing for surgical resection for CPAM is within the first year of life to reduce infection risks and facilitate technical feasibility, despite the small size of the patients [19]. Genetic counseling and detailed histopathological examination are vital, particularly considering the potential for DICER1 mutations. Another interesting study done by Hung et al. explored long‐term complications after surgical resection of CLM. Long‐term complications after surgery were observed in 73.7% of patients, including chronic cough, recurrent lower airway infections, wheezing, poor exercise tolerance, and orthopedic complications. Interestingly, some lesions can also regress or reduce in size during pregnancy, even up to 31% of cases, but also increase in size up to 8.5% of cases [20]. Neonatal morbidity was observed in 20.6% of newborns with CLM, with 46% undergoing elective surgery. In cases with hydrops, fetal or perinatal loss was 42%. The role of fetal therapy in improving outcomes is still unclear due to the small number of cases and intervention heterogeneity [20].

Finally, in all our patients, the prognosis was good, which is in line with other studies showing that mortality rates are low, even for symptomatic patients [21].

## 6 Conclusion

The study highlights the variability in age and symptoms at diagnosis for CLMs, emphasizing the need for individualized management. Pulmonary sequestration cases typically presented at an older age compared to CPAM, due to less pronounced early‐life symptoms. Surgical intervention is crucial in symptomatic cases, particularly for CPAM, due to the risk of malignant transformation.

Collaborative management involving obstetricians, neonatologists, surgeons, oncologists, and geneticists is essential for optimal outcomes. Early diagnosis and intervention are critical for improving prognosis in children with CLMs.

## Author Contributions

Conceptualization, P.S.‐S., A.K., P.M., I. W.‐B. and D. J.‐L.; methodology, P.S.‐S., A.K., P.M., I. W.‐B. and D. J.‐L.; software, P.S.‐S., A.K., P.M., I. W.‐B. and D. J.‐L.; validation, P.S.‐S., A.K., P.M., I. W.‐B.and D. J.‐L.; formal analysis, P.S.‐S., A.K., I. W.‐B. and D. J.‐L.; investigation, P.S.‐S., A.K., I. W.‐B. and D. J.‐L.; resources, P.S.‐S., A.K., P.M., I. W.‐B. and D. J.‐L.; data curation, P.S.‐S., A.K., P.M., I. W.‐B. and D. J.‐L.; writing—original draft preparation, P.S.‐S. and A.K.; writing—review and editing, P.M., I. W.‐B. and D. J.‐L.; visualization, P.S.‐S., A.K., P.M., I. W.‐B. and D. J.‐L..; supervision, D. J.‐L.; project administration, P.M., I.W.‐B. and D. J.‐L.; funding acquisition, P.S.‐S., A.K., P.M., I. W.‐B. and D.J.‐L. All authors have read and agreed to the published version of the manuscript.

## Funding

The authors have nothing to report.

## Ethics Statement

The study was conducted in accordance with the declaration of Helsinki, and approved by the Bioethics Committee of the Poznan University of Medical Sciences (Resolution no. KB‐280/24, 5 April 2024).

## Conflicts of Interest

The authors declare no conflicts of interest.

## Data Availability

The data that support the findings of this study are available on request from the corresponding author. The data are not publicly available due to privacy or ethical restrictions.
